# Mast cell tryptase and asthma

**DOI:** 10.1080/09629359791433

**Published:** 1997-12

**Authors:** M-Q. Zhang, H. Timmerman

**Affiliations:** 1 Division of Medicinal Chemistry Leiden/Amsterdam Centre for Drug Research Vrije Universiteit De Boelelaan 1083 Amsterdam 1081 HV The Netherlands; 2 Scientific Development Group Organon Laboratories Ltd Newhouse Lanarkshire ML1 5SH UK

## Abstract

Recent physiological and pharmacological studies have indicated the potential importance of tryptase, the major protein component in mast cells, in inflammatory diseases (especially asthma). Being released at inflammatory sites after the activation of mast cells, tryptase is capable of causing bronchohyperresponsiveness and infiltration of eosinophils, neutrophils, etc. in animal airways. The mechanisms by which tryptase causes bronchoconstriction involve probably the potentiation of other chemical mediators such as histamine, production of bradykinin via the hydrolysis of kininogen, and cleavage of the bronchodilating peptides VIP (vasoactive intestinal peptide) and PHM (peptide histidine-methionine). Tryptase has also been found to be a potent mitogen *in vitro* for airway smooth muscle cells and epithelial cells, implying its role in the hyperplasia of the asthmatic airways. The experimental data providing evidence for the above roles of tryptase are summarized in the present review, as well as the effects of tryptase inhibition in animal asthma models. The potential strategies for the development of anti-asthmatic agents based on the inhibition of tryptase are discussed.


Mediators of Inflammation, 6, 311–317 (1997)

### Mast cell tryptase and asthma

M-Q. Zhang[CA,*] and H. Timmerman

Division of Medicinal Chemistry, Leiden/Amsterdam Centre for Drug Research, Vrije Universiteit, De Boelelaan 1083, 1081 HV Amsterdam, The Netherlands
*Present address: Scientific Development Group, Organon Laboratories Ltd, Newhouse, Lanarkshire ML1 5SH, UK

[CA,*]Corresponding Author and Address
Tel: (+44) 1698 732611
Fax: (+44) 1698 733252

**Recent physiological and pharmacological studies have indicated the potential importance of tryptase, the major protein component in mast cells, in inflammatory diseases (especially asthma). Being released at inflammatory sites after the activation of mast cells, tryptase is capable of causing bronchohyperresponsiveness and infiltration of eosinophils, neutrophils, etc. in animal airways. The mechanisms by which tryptase causes bronchoconstriction involve probably the potentiation of other chemical mediators such as histamine, production of bradykinin via the hydrolysis of kininogen, and cleavage of the bronchodilating peptides VIP (vasoactive intestinal peptide) and PHM (peptide histidine-methionine). Tryptase has also been found to be a potent mitogen *in vitro* for airway smooth muscle cells and epithelial cells, implying its role in the hyperplasia of the asthmatic airways. The experimental data providing evidence for the above roles of tryptase are summarized in the present review, as well as the effects of tryptase inhibition in animal asthma models. The potential strategies for the development of anti-asthmatic agents based on the inhibition of tryptase are discussed.**

**Key words:** $H_1$-antagonists, Asthma, Histamine, Mast cells, Tryptase, Tryptase inhibitors

### Introduction

Mast cells have long been implicated in the pathogenesis of asthma, particularly in the acute response occurring immediately after exposure to allergen. Binding of allergen molecules to IgE antibodies on mast cells and the subsequent cross-linking directly or indirectly activate various enzymes in the cell membrane. Cascades involving tyrosine kinase enzymes, phospholipase C, protein kinase C and an influx of calcium ions induce chemical-laden granules to release their preformed contents. These cascades also appear to promote the synthesis and extrusion of lipid chemicals such as prostaglandins and leukotrienes. The various chemicals emitted by active mast cells may induce many allergic symptoms as well as the migration of eosinophils, basophils and other cells into the tissue so as to cause inflammation.

Histamine is perhaps the most infamous of those preformed chemical mediators released by mast cells, and antihistamines have thus attracted a great deal of research attention.[1] Much less is known, however, about the serine proteases, tryptase and chymase that are also preformed in the secretory granules of mast cells. Together these enzymes represent the major protein components of the mast cell, up to 20–25% of the total protein content.[2] Tryptase is particularly notable for its abundance in mast cells. It resides in the secretory granules of all mast cells from the time granules first begin to form,[3] whereas others dwell in the granules of a subpopulation of mast cells. Those mast cells with tryptase only are called $MC_T$ cells, and those with all of the enzymes are called $MC_{TC}$ cells.[4] $MC_T$ and $MC_{TC}$ cells have quite different sets of distribution in human tissues. Whereas $MC_T$ cells are the major component in alveolar tissue and small intestine mucosa, mast cells in skin and ocular conjuctiva are mostly $MC_{TC}$ cells. At present no physiological or pathological function has been definitively assigned to mast cell tryptase although it has been used as a specific marker of mast cell activation.[5] The abundance of the enzyme in mast cells, in conjunction with some of its recently observed biological activities, warrants further research on the potential role of tryptase in allergy and inflammation. In this review, the current knowledge of molecular properties and biological activities of tryptase, specially those related to asthma, will be presented together with the effect of tryptase inhibition in animal asthma models.

© 1997 Rapid Science Publishers

### Biochemistry of Mast Cell Tryptase

### Molecular properties of tryptase

Mast cell tryptase (EC 3.4.21.59) is a trypsin-like serine proteinase with 40% sequence identity with trypsin and only 20 or 21 additional residues. However unlike trypsin, tryptase only cleaves a limited number of proteins and is not inhibited by endogenous proteinase inhibitors such as α1-proteinase inhibitors and secretory leukocyte protease inhibitors.[6,7] Several different sequences for human tryptase have been reported and a few examples are listed in Table 1. They are highly conserved (> 70% homology) and all form homogenous tetramers of a total molecular mass of 110–150 kDa, with each subunit of 31 to 38 kDa (Table 1). A reduction in molecular mass of tryptase subunits of 2000–4000 Da after treatment with endoglycosidase indicates that carbohydrates are present on each subunit.[10] Inside mast cell granules and after secretion, tryptase is stabilized in its active tetrameric form by binding to heparin, via ionic interactions.[8] When free in solution, tryptase subunits irreversibly dissociate from one another into inactive monomers, without any evidence for autodegradation. Substantial conformational changes occur during this process, as evidenced by circular dichroic spectral shifts and by distinct epitopes being detected on the active tetramer and inactive monomers.[12]

### 3D structures of tryptase

The three-dimensional structures of tryptase have been predicted by molecular modelling based on the crystal structure of bovine trypsin.[13] The models show two large insertions to lie on either side of the active-site cleft, suggesting an explanation for the limited activity of tryptases on protein substrates and the lack of inhibition by natural inhibitors. A group of conserved Trp residues and a unique proline-rich region make two surface hydrophobic patches that may account for the formation of tetramers and/or inhibition with increasing salt. Although they contain no consensus heparin-binding sequence, the tryptases have 10–13 more His residues than trypsin, and these are positioned on the surface of the model. In addition, clustering of Arg and Lys residues may also contribute to heparin binding. Putative Asn-linked glycosylation sites are found on the opposite side of the model from the active site. The model provides structural explanations for some of the unusual characteristics of the tryptases and a rational basis for future experiments, such as site-directed mutagenesis.

Recently five different tryptase cDNA molecules have been cloned and sequenced, two (α and β) from human lung mast cell cDNA library and three (I, II, III) from human skin mast cell library.[14] Two genes for human tryptase have been localized to chromosome 16.[15,16] One corresponds to α-tryptase and tryptase III, and the other to β-tryptase, tryptase I, and tryptase II. The amino acid sequence of α-tryptase contains 24 amino acid differences and a single amino acid deletion compared to β-tryptase sequence. These two isoforms of human lung tryptase were reported to have different cleavage rates for peptide substrates, e.g. VIP.[17]

Besides human tryptases, animal tryptases have also been purified. These include guinea-pig lung tryptase,[18] bovine tryptase,[19] dog tryptase,[20] rat tryptase,[21] murine tryptase,[22] and gerbil tryptase.[23]

### Enzymatic assays of tryptase activity

Tryptase activity is typically measured by the rate of cleavage of small synthetic ester or peptide substrates with Arg or Lys in the C-terminal position. An example is given as follows.[14] To a solution of 1 mM tosyl-L-arginine methyl ester (TAME) in 1 ml 0.04 M Tris-HCl buffer (pH 8.1) containing 0.15 M NaCl is added tryptase at either room temperature or at 37°C. The molar extinction coefficient for the change in absorbance at 247 nm equals 540. The concentration of TAME can be confirmed by measuring the absorbance at 247 nm after complete cleavage of the substrate by excess trypsin. Purified tryptase typically exhibits a specific activity of 100–120 U/mg.

A variety of tripeptide derivatives of *p*-nitroanilide are also used to assay tryptase.[24] The

**Table 1.** Molecular properties of human tryptase from difference sources

| Source | Molecular weight (heparin) | No. of subunit | Subunit molecular weight (kDa) | Glycosylation | Reference |
|---|---|---|---|---|---|
| Lung | 130 (+) | 4 | 31–33/35–37 | + | 2, 6, 8 |
| Skin | 120 (+) | 4 | 34/38 | + | 9 |
| Pituitary | 110 (?) | 4 | 34/36 | + | 10 |
| HMC-1 | 150 (+) | 4 | 32/35 | + | 11 |

change in absorbance of *p*-nitroaniline at 405 nm is used as a indicator for the cleavage.

### Mast Cell Tryptase in Asthma

### Elevated levels of tryptase in asthma

Tryptase resides in secretory granules of human mast cells and is released together with histamine upon the actions of certain stimuli. Histamine, once released, is free to diffuse rapidly through tissue into the blood stream or onto tissue surfaces, whereas tryptase diffuses more slowly, presumably because of its macromolecular complex. During bee-sting induced anaphylaxis, tryptase levels in the blood stream are maximal at 60–120 min, whereas those of histamine are maximal at 5 min.[25] Tryptase levels then decline with a half-life of 1.5–2.5 h, and histamine with a half-life of only a few minutes. In skin chamber fluid[26] and surface of airway,[27] the analogous but shorter delay of tryptase appearance in comparison with histamine were also reported. Because of the association of mast cell activation with many allergic diseases, it is not surprising to observe elevated levels of tryptase in these allergic conditions. Several studies have shown that tryptase levels in asthmatic patients are significantly higher than those in normal subjects.[27] Thus allergen challenge in atopic subjects caused an elevation in tryptase levels in bronchoalveolar lavage fluid above baseline values both in asthmatic and nonasthmatic subjects, but not in nonatopic groups. In aspirin-sensitive asthmatics, the increase in nasal symptoms after aspirin ingestion was found to correlate with the increase in nasal tryptase levels (mean maximal increase: $3{\cdot}5 \pm 26$ ng/ml, versus placebo: $0{\cdot}1 \pm 0{\cdot}2$ ng/ml, $P < 0{\cdot}05$) as well as the increase of nasal histamine and leukotriene levels.[28] Interestingly treatment with zileuton, a 5-LO inhibitor, blocked the increase in nasal symptoms after aspirin ingestion, and it also blocked the rise in nasal tryptase ($P = 0{\cdot}011$) and nasal leukotriene ($P < 0{\cdot}05$) levels, but not nasal histamine. Tryptase is also implicated in adenosine (AMP)-induced bronchoconstriction.[29] Immediately after instillation of AMP directly into an airway segment of eight asthmatic subjects, a prompt reduction in airway caliber was observed in parallel with a significant rise in $PGD_2$, histamine, and tryptase levels in the lavage fluid. After AMP challenge, the median (range) concentration for tryptase was changed from 0.30 to 0.54 ng/ml ($P = 0{\cdot}013$). These results indicate that adenosine-induced responses may be initiated by the acute release of mast-cell-derived mediators, including $PGD_2$, histamine, and tryptase.

The presence of higher tryptase ($10 \pm 7$ U/l vs. $0{\cdot}9 \pm 0{\cdot}9$ U/l, $P = 0{\cdot}0001$) levels was also reported in patients with acute severe asthma than in those with cystic fibrosis.[30]

### Biological activities of tryptase relevant to asthma

Although the biological activities of tryptase have not been clearly elucidated, a number of actions of tryptase potentially relevant to asthma have been reported (Fig. 1).

Tryptase appears to potentiate the action of histamine and other contracting agonists so that to cause smooth muscle hyperresponsiveness in asthma.[31] Preincubation of dog tracheal smooth muscle with tryptase *in vitro* caused a marked leftward shift of the histamine concentration–response curve with the $ED_{50}$ of histamine decreasing from 19 μM to 2 μM (Fig. 2a). The maximum tension induced by histamine after the treatment with tryptase was increased from 110 to 170 g/g tissue weight, but tryptase alone did not increase resting tension. These augmented contractile responses to histamine were dependent on the concentration of tryptase added (Fig. 2b), and they were reversed by a histamine $H_1$ antagonist or prevented by a tryptase inhibitor.

Similar effects of tryptase to the contractile potency of serotonin and KCl, but not acetylcholine, were also observed, suggesting that the enzyme exerts its effects at a point in the stimulus-contraction pathway shared by the three chemically distinct agonists. Because the smooth muscle effects of histamine, serotonin and KCl depend on the movement of external $Ca^{2+}$ into the cell, and because $Ca^{2+}$ channel blockers (nifedipine and verapamil) abolish

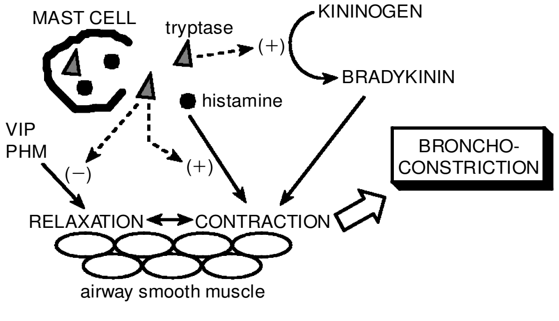

FIG. 1. Pathogenetic activities of tryptase in asthma. Tryptase augments the contractile potency of other chemical mediators, e.g. histamine; hydrolyses kininogen to produce bradykinin, and cleaves the bronchodilators vasoactive intestinal peptide (VIP) and peptide histidine-methionine (PHM). (See text for details.)

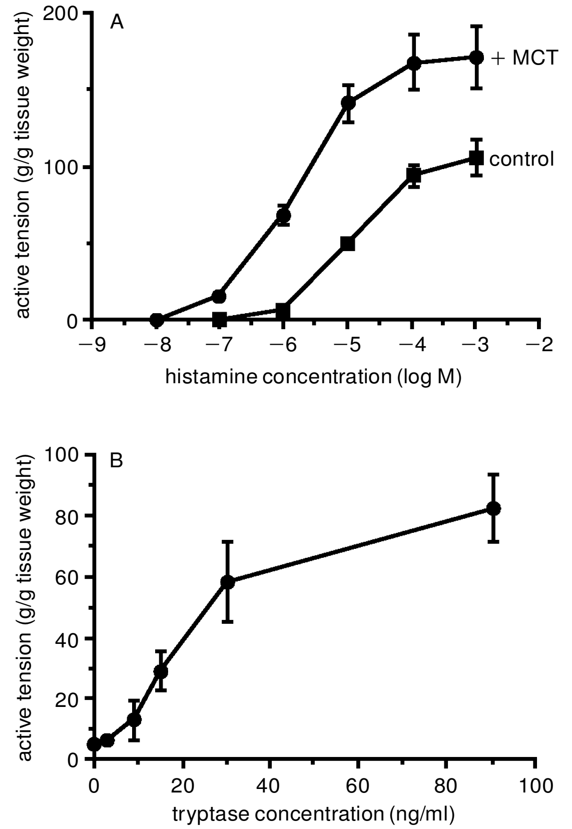

FIG. 2. (A) Effect of tryptase (90 ng/ml) on concentration–response curve of histamine in dog bronchial smooth muscle; (B) concentration-dependency of tryptase on histamine (1 μM) induced contraction in dog bronchial rings (made according to Sekizawa *et al.*[31]).

contractions induced by the combination of tryptase and histamine, it was speculated that tryptase might affect airway smooth muscle responsiveness by modifying $Ca^{2+}$ channels. This hypothesis is supported by the failure of tryptase to augment the contractions produced by acetylcholine, an agonist whose smooth muscle effects are mediated by a mechanism independent of voltage-dependent $Ca^{2+}$ channels. Tryptase could affect the channel itself or cleave a regulatory protein on the cell surface.

Recently in a study of 17 children with mild to moderately severe chronic asthma,[32] bronchial responsiveness to histamine was found to correlate highly with mast cell tryptase levels ($r = -0{\cdot}714$, $P < 0{\cdot}005$), further supporting the notion that tryptase possibly induced upregulation of bronchial smooth muscle tone.

Mast cells have long been suggested to play a role in the modulation of neuropeptides because of its close anatomic association with peptidergic nerves. It was reported that human tryptase rapidly hydrolysed vasoactive intestinal peptide (VIP) and peptide histidine-methionine (PHM), but not the tachykinin substance P.[33] VIP and PHM are potent bronchodilating peptides co-transcribed and co-expressed in airway motor neurones. Degradation of these bronchodilating peptides in connection with the unchanged level of the bronchoconstricting substance P may contribute to the increase in bronchial responsiveness associated with asthma. *In vitro* animal studies have indeed showed that tryptase reverse VIP-induced airway smooth muscle relaxation of guinea-pig lung[34] and dog trachea.[35]

Tryptase has also been reported to hydrolyse kininogen to produce bradykinin,[36,37] a nonapeptide which is one of the most potent vasodilators and increases vascular permeability. It is known that kininogen is consumed during anaphylactic reactions in man, and consequently bradykinin and lysylbradykinin are generated.[38] Administration of bradykinin to the lower airways induces bronchoconstriction in asthmatics. Under optimal conditions (pH 5.5), human tryptase produced bradykinin from kininogen at a rate of 10–12 μg bradykinin/h/mg, but only 2 μg bradykinin/h/mg at pH 7.2.[36] Since the optimal pH is within the range of mast cell granule, it is also possible that this activity of tryptase is retained to another intracellular substrate. Tryptase additionally cleaves C3 and generates the anaphylatoxin, C3a, a potent bronchospastic agent.[14]

Tryptase has further been shown to be a potent mitogen for airway smooth muscle cells[39,40] and epithelial cells.[41] At a concentration of 4 nM, tryptase increased dog tracheal smooth muscle cell numbers 2.1- and 2.8-fold above controls after 2 or 4 days of incubation, respectively.[39] These increases were approximately the same as those induced by platelet-derived growth factor (50 ng/ml) or 10% calf serum. With respect to potency, tryptase caused concentration-dependent increases in bromodeoxyuridine (BrdU) uptake, as detected in an enzyme-linked immunosorbent assay or by counting BrdU-labelled nuclei, with an $EC_{50}$ of 2 nM. The mitogenic effect of tryptase for airway smooth muscle cells could be responsible for the hyperplasia in the airways of asthmatic patients and may contribute to the development of the bronchial hyperresponsiveness that occurs in these patients.

Tryptase was also found to stimulate DNA synthesis in the human epithelial cells *in vitro*.[41] Maximal growth of the human epithelial cell line H292 was observed after 24 h using 25 mU/ml of tryptase, a concentration that is likely to be achieved *in vivo*. Inhibitors of tryptase activity, including leupeptin and benzamidine hydrochloride, significantly decreased tryptase-induced stimulation of DNA synthesis, indicating the requirement for an active catalytic site. Tryptase also stimulated a catalytic site-dependent release of IL-8 from epithelial cells after 24 h, and this was associated with upregulation of ICAM-1 expression, as revealed by FACS analysis. Tryptase may thus play a critical role in epithelial repair and in the recruitment of granulocytes following mast cell activation. Indeed, it has recently been shown that tryptase acted as a chemoattractant for eosinophils and neutrophils, and could activate eosinophils and mast cells.[42] Since eosinophils are likely the most important inflammatory cells in asthma, the recruitment of eosinophils may account for the potential inflammatory activities of tryptase.

### Inhibitors of Mast Cell Tryptase as Anti-asthmatic Agents

### Inhibition of tryptase release

Tryptase is released together with histamine from mast cells, and therefore those mast cell membrane stabilizers, which inhibit the release of histamine, should also be able to inhibit the release of tryptase. Indeed oxatomide, a histamine $H_1$ receptor antagonist known to inhibit histamine release from mast cells, is also effective in the inhibition of tryptase release.[43] Preincubation (15 min, 37°C) of HLMC and HSMC cells with oxatomide ($10^{-7}$–$10^{-5}$ M) before anti-IgE challenge concentration-dependently (10–40%) inhibited the immunologic release of histamine, tryptase and $LTC_4$, indicating that oxatomide exerts anti-inflammatory activities by inhibiting the release of preformed and *de novo* synthesized mediators from human mast cells and basophils.

Loratadine, another histamine $H_1$ receptor antagonist relatively effective in the treatment of bronchial asthma, was shown to inhibit the exudation of $\alpha_2$-macroglobulin and to reduce tryptase levels.[44] The reduction of tryptase levels in nasal lavage fluid by loratadine correlated with its reduction of nasal symptoms and obstruction in allergic rhinitis. The inhibitory effects of loratadine on nasal lavage fluid levels of $\alpha_2$-macroglobulin suggest that histamine, through effects on microvascular $H_1$-receptors, mediates allergen-induced exudation of bulk plasma in acute allergic rhinitis. The reduced lavage fluid levels of tryptase suggest either that loratadine directly attenuates mast cell release activity or that loratadine, through inhibition of the exudation process, simply attenuates luminal entry of tryptase.

### Inhibition of tryptase effectors

As discussed earlier, tryptase exerts its biological activity via a number of effectors, e.g. histamine, bradykinin, etc. Therefore, inhibition of tryptase effectors would also in principle limit the effects of tryptase. It has been shown that inhaled tryptase (100 and 500 ng in 2 ml $H_2O$ solution) increased pulmonary flow resistance (RL) (mean ± SE) by 33 ± 12 and 122 ± 8% ($P < 0{\cdot}05$) over baseline.[45] The response was reproducible upon repeat challenges. The response to tryptase was blocked by pretreating the sheep intravenously with the histamine $H_1$ antagonist chlorpheniramine (2 mg/kg), in which RL increased only 5 ± 4 and 7 ± 6% after 700 and 500 ng tryptase.[45]

It has also been demonstrated that intradermal injection of tryptase-heparin complex (1 or 10 ng tryptase + 3 U heparin) in allergic sheep caused immediate cutaneous reaction (ICR) 50–82% relative to that caused by histamine (5% wt/vol).[46] This response is specific because tryptase and heparin alone caused only minimal ICR, ruling out the possibility that the animals had become sensitized to the tryptase or some other unknown proteins. Furthermore a mixture of heat-inactivated tryptase and heparin also caused no ICR. In addition to the blockade by tryptase inhibitors, the ICR caused by tryptase–heparin complex can also be blocked by a combination of histamine $H_1$ and $H_2$ antagonists [chlorpheniramine (2 mg/kg) + metiamide (3 mg/kg)], indicating that tryptase acts via histamine.[46] One possible mechanism may be that tryptase regulates mediator release from mast cells.[42]

### Inhibition of tryptase activity

The most direct proof of tryptase's role in asthma comes from the selective inhibition of tryptase itself. APC 366 (Fig. 3), a selective tryptase inhibitor discovered by Arris Pharmaceutical Corporation, has been shown to block antigen-induced bronchoconstriction and inflammatory responses in allergic sheep.[45,47] APC 366 (apparent Ki = 330 nM) is a selective

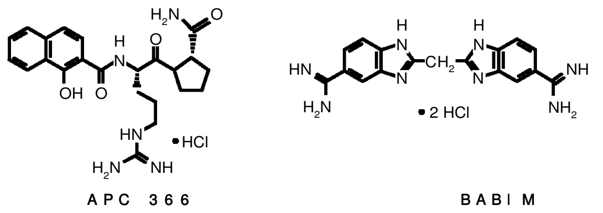

FIG. 3. Structures of tryptase inhibitors APC 366 and BABIM.

tryptase inhibitor without significant activity against other proteases. Acute treatment (0.5 h before challenge) with APC 366 (9 mg/3 ml $H_2O$, aerosol) did not affect the maximum bronchoconstriction of allergic sheep immediately after challenge but did cause a more rapid reversal of the increase in specific lung resistance. This response was associated with a significant inhibition of tryptic activity in the bronchoalveolar lavage fluid. Twenty-four hours after challenge, APC 366 completely blocked the antigen-induced airway hyperresponsiveness to inhaled carbachol observed in the control experiments, but did not block histamine-induced bronchoconstriction.

When given 18 mg twice daily for 3 days and once 18 mg again 30 min before antigen challenge, APC 366 markedly inhibited the immediate response as compared with the acute treatment. The marked inhibition of the immediate bronchial response to antigen under this prophylactic treatment could indicate that tryptase is not only released in conjunction with other preformed mast cell mediators but might also act to modulate the release and/or effectiveness of the other spasmogens. APC 366 also showed anti-inflammatory actions by blocking antigen-induced eosinophil accumulation in bronchoalveolar lavage fluid (treatment: 1.6 to 5.1 eosinophils/$mm^2$ vs. control: 1.2 to 17.2 eosinophils/$mm^2$, $P < 0{\cdot}05$), as well as the increase of albumin levels in bronchoalveolar lavage fluid. These results further support the suggestion that tryptase may act as a chemoattractant to eosinophils[42] and hydrolyse kininogen to bradykinin so to increase vascular permeability.[36,37]

Similar anti-asthmatic activity has been demonstrated with BABIM (Fig. 3), another potent and reversible inhibitor of tryptase (Ki = 1·8 nM).[47] A limited structure-activity relationship study showed that both amidine groups of BABIM are important for potent tryptase inhibitory activity, and at least one of the amidine groups should be unsubstituted since cyclization of both amidine groups into imidazoline rings afforded a compound without activity.[48] BABIM was shown to block completely the reversal of VIP-induced relaxation of isolated trachea by dog tryptase.[48]

### General Remarks

Because tryptase is unique to mast cells (although small amount of tryptase have been detected in basophils), inhibition of tryptase offers potential selectivity towards the conditions where mast cells are predominant. There is compelling evidence to suggest the important role of mast cells in allergic asthma. The limited success of anti-asthmatic agents related to actions on mast cells, e.g. antihistamines and DSCG, may be attributed to the low airway tissue concentrations achievable with systematic application of these agents. It has been reported that cetirizine, a non-sedating $H_1$ antagonist, when given orally 10 mg twice daily for 1 week, failed to inhibit exercise induced bronchoconstriction (maximum falls in $FEV_1$ 28% and 27% of baseline), but when given by inhalation (1 ml, 5 and 10 mg/ml) cetirizine markedly reduced the fall in $FEV_1$ after exercise by 15.2%, and 10.2% of baseline, respectively, compared with 23.7% after placebo.[49] Tryptase inhibitors may have potentially a similar fate when applied in the treatment of asthma. However, the inflammatory actions of tryptase, e.g. recruitment of eosinophil, synthesis of bradykinin, etc., may afford tryptase inhibitors additional anti-inflammatory profiles as seen in the animal studies of APC 366. These anti-inflammatory activities coupled with symptomatic relieving effects of tryptase inhibitors should warrant them as promising candidates for further research and development. Recent phase II clinical trials results of APC 366 have showed that the agent at doses of 2.5 or 5.0 mg three times a day for a total of 13 doses improved both the late and the hyperresponsive phase of the asthmatic reaction to allergen, compared to baseline. The full scientific report of this study and many more advanced clinical trials is eagerly awaited. By then, the anti-asthmatic advantage of tryptase inhibitors would have been more evident.

### References

1. Zhang MQ, Leurs R, Timmerman H. Histamine $H_1$ receptor antagonists. In: Wolff ME (ed.) *Burger's Medicinal Chemistry and Drug Discovery* (5th Ed), Vol. 5: Therapeutic Agents. New York: John Wiley, 1997; 495–559.
2. Schwartz LB, Lewis RA, Austen KF. Tryptase from human pulmonary mast cells. Purification and characterization. *J Biol Chem* 1981; **256**: 11939–11943.
3. Craig SS, Scheimer NM, Schwartz LB. Ultrastructural analysis of maturing human T and TC mast cells in situ. *Lab Invest* 1989; **60**: 147–157.
4. Irani AA, Scheimer NM, Craig SS, DeBlois G, Schwartz LB. Two types of human mast cells that have distinct neutral protease compositions. *Proc Natl Acad Sci USA* 1986; **83**: 4464–4468.
5. Schwartz LB. Laboratory assessment of immediate hypersensitivity and anaphylaxis—use of tryptase as marker of mast cell-dependent events. *Immunol Allergy Clin NAm* 1994; **14**: 339–349.
6. Smith TJ, Hougland MW, Johnson DA. Human lung tryptase. Purification and characterization. *J Biol Chem* 1984; **259**: 11046–11051.
7. Alter SC, Kramps JA, Janoff AA, Schwartz LB. Interactions of human mast cell tryptase with biological protease inhibitors. *Arch Biochem Biophys* 1990; **276**: 26–31.
8. Schwartz LB, Bradford TR. Regulation of tryptase from human lung mast cells by heparin. Stabilization of the active tetramer. *J Biol Chem* 1986; **261**: 7372–7379.
9. Harvima IT, Schechter NM, Harvima RJ, Fräki JE. Human skin tryptase: purification, partial characterization and comparison with human lung tryptase. *Biochim Biophys Acta* 1988; **957**: 71–80.

10. Cromlish JA, Siedah NG, Marcinkiewicz M, Hamelinm J, Johnson DA, Chretein M. Human pituitary tryptase: molecular forms, $NH_2$-terminal sequence, immuno-cytochemical localization, and specificity with prohormone and fluorogenic substrates. *J Biol Chem* 1987; **262**: 1363–1373.
11. Butterfield JH, Weiler DA, Hunt LW, Wynn SR, Roche PC. Purification of tryptase from a human mast cell line. *J Leukocyte Biol* 1990; **47**: 409–419.
12. Schwartz LB, Bradford TR, Lee DC, Chlebowski JF. Immunologic and physicochemical evidence for conformational changes occurring on conversion of human mast cell tryptase from active tetramer to inactive monomer. Production of monoclonal antibodies recognizing active tryptase. *J Immunol* 1990; **144**: 2304–2311.
13. Johnson DA, Barton GJ. Mast cell tryptases: examination of unusual characteristics by multiple sequence alignment and molecular modeling. *Protein Sci* 1992; **1**: 370–373.
14. Schwartz LB. Tryptase: a mast cell serine protease. *Meth Enzymol* 1994; **244**: 88–100.
15. Miller JS, Moxley G, Schwartz LB. Cloning and characterization of a second complementary DNA for human tryptase. *J Clin Invest* 1990; **86**: 864–870.
16. Vanderslice P, Ballinger SM, Tam EK, Goldstein SM, Craik CS, Caughey GH. Human mast cell tryptase: multiple cDNAs and genes reveal a multigene serine protease family. *Proc Natl Acad Sci USA* 1990; **87**: 3811–3815.
17. Little SS, Johnson DA. Human mast cell tryptase isoforms: separation and examination of substrate-specificity differences. *Biochem J* 1995; **307**: 341–346.
18. McEuen AR, He S, Brander ML, Walls AF. Guinea pig lung tryptase. Localisation to mast cells and characterisation of the partially purified enzyme. *Biochem Pharmacol* 1996; **52**: 331–340.
19. Pallaoro M, Gambacurta A, Fiorucci L, Mignogna G, Barra D, Ascoli F. cDNA cloning and primary structure of tryptase from bovine mast cells, and evidence for the expression of bovine pancreatic trypsin inhibitor mRNA in the same cells. *Eur J Biochem* 1996; **237**: 100–105.
20. Vanderslice P, Craik CS, Nadel JA, Caughey GH. Molecular cloning of dog mast cell tryptase and a related protease: structural evidence of a unique mode of serine protease activation. *Biochemistry* 1989; **28**: 4148–4155.
21. Braganza VJ, Simmons WH. Tryptase from rat skin: purification and properties. *Biochemistry* 1991; **30**: 4997–5007.
22. Chu W, Johnson DA, Musich PR. Molecular cloning and characterization of mouse mast cell chymases. *Biochim Biophys Acta* 1992; **1121**: 83–87.
23. Murakumo Y, Ide H, Itoh H, *et al.* Cloning of the cDNA encoding mast cell tryptase of Mongolian gerbil, Meriones unguiculatus, and its preferential expression in the intestinal mucosa. *Biochem J* 1995; **309**: 921–926.
24. Tanaka T, McRae BJ, Cho K, *et al.* Mammalian tissue trypsin-like enzymes. Comparative reactivities of human skin tryptase, human lung tryptase, and bovine trypsin with peptide 4-nitroanilide and thioester substrates. *J Biol Chem* 1983; **258**: 13552–13557.
25. Schwartz LB, Yumginger JW, Miller JS, Bokhari R, Dull D. The time course of appearance and disappearance of human mast cell tryptase in the circulation after anaphylaxis. *J Clin Invest* 1989; **83**: 1551–1555.
26. Tsubaki T, Iikura Y, Saito H, *et al.* Changes in histamine/tryptase levels in skin chambers—application for clinical evaluation of atopic dermatitis. *Int Arch Allergy Immunol* 1992; **99**: 459–462.
27. Schwartz LB. Cellular inflammation in asthma: neutral proteases of mast cells. *Am Rev Respir Dis* 1992; **145**: S18–S21.
28. Fischer AR, Rosenberg MA, Lilly CM, *et al.* Direct evidence for a role of the mast cell in the nasal response to aspirin in aspirin-sensitive asthma. *J Allergy Clin Immunol* 1994; **94**: 1046–1056.
29. Polosa R, Ng WH, Crimi N, *et al.* Release of mast-cell-derived mediators after endobronchial adenosine challenge in asthma. *Am J Resp Crit Care Med* 1995; **151**: 624–629.
30. Fahy JV, Kim KW, Liu J, Boushey HA. Prominent neutrophilic inflammation in sputum from subjects with asthma exacerbation. *J Allergy Clin Immunol* 1995; **95**: 843–852.
31. Sekizawa K, Caughey GH, Lazarus SC, Gold WM, Nadel JA. Mast cell tryptase causes airway smooth muscle hyperresponsiveness in dogs. *J Clin Invest* 1989; **83**: 175–179.
32. Ferguson AC, Whitelaw M, Brown H. Correlation of bronchial eosinophil and mast cell activation with bronchial hyperresponsiveness in children with asthma. *J Allergy Clin Immunol* 1992; **90**: 609–613.
33. Tam EK, Caughey GH. Degradation of airway neuropeptides by human lung tryptase. *Am J Respir Cell Mol Biol* 1990; **3**: 27–32.
34. Lilly CM, Martins MA, Drazen JM. Peptidase modulation of vasoactive intestinal peptide pulmonary relaxation in tracheal superfused guinea pig lungs. *J Clin Invest* 1993; **91**: 235–243.
35. Franconi GM, Graf PD, Lazarus SC, Nadel JA, Caughey GH. Mast cell tryptase and chymase reverse airway smooth muscle relaxation induced by vasoactive intestinal peptide in the ferret. *J Pharmacol Exp Ther* 1989; **24**: 133–137.
36. Proud D, Siekierski ES, Bailey GS. Identification of human lung mast cell kininogenase as tryptase and relevance of tryptase kininogenase activity. *Biochem Pharmacol* 1988; **37**: 1473–1480.
37. Walls AF, Bennett AR, Sueiras-Diaz J, Olsson H. The kininogenase activity of human mast cell tryptase. *Biochem Soc Transaction* 1992; **20**: 260s.
38. Smith PL, Kagey-Sobotka A, Bleecker ER, *et al.* Physiologic manifestations of human anaphylaxis. *J Clin Invest* 1980; **66**: 1072–1080.
39. Brown JK, Tyler CL, Jones CA, Ruoss SJ, Hartmann T, Caughey GH. Tryptase, the dominant secretory granular protein in human mast cells, is a potent mitogen for cultured dog tracheal smooth muscle cells. *Am J Respir Cell Mol Biol* 1995; **13**: 227–236.
40. Brown JK, Tyler CL, Jones CA, Ruoss SJ, Hartmann T, Caughey GH. Tryptase-induced mitogenesis in airway smooth muscle cells. Potency, mechanisms, and interactions with other mast cell mediators. *Chest* 1995; **107**: 95S–96S.
41. Cairns JA, Walls AF. Mast cell tryptase is a mitogen for epithelial cells. Stimulation of IL-8 production and intercellular adhesion molecule-1 expression. *J Immunol* 1996; **156**: 275–283.
42. Walls AF, He S, Teran LM, *et al.* Granulocyte recruitment by human mast cell tryptase. *Int Arch Allergy Immunol* 1995; **107**: 372–373.
43. Patella V, de Crescenzo G, Marino O, Spadaro G, Genovese A, Marone G. Oxatomide inhibits the release of proinflammatory mediators from human basophils and mast cells. *Int Arch Allergy Immunol* 1996; **111**: 23–29.
44. Greiff L, Persson CGA, Svensson C, Enander I, Andersson M. Loratadine reduces allergen-induced mucosal output of alpha(2)-macroglobulin and tryptase in allergic rhinitis. *J Allergy Clin Immunol* 1995; **96**: 97–103.
45. Molinari JF, Scuri M, Moore WR, Clark J, Tanaka R, Abraham WM. Inhaled tryptase causes bronchoconstriction in sheep via histamine release. *Am J Respir Crit Care Med* 1996; **154**: 649–653.
46. Molinari JF, Moore WR, Clark J, Tanaka R, Butterfield JH, Abraham WM. Role of tryptase in immediate cutaneous responses in allergic sheep. *J Appl Physiol* 1995; **79**: 1966–1970.
47. Clark JM, Abraham WM, Fishman CE, *et al.* Tryptase inhibitors block allergen-induced airway and inflammatory responses in allergic sheep. *Am J Respir Crit Care Med* 1995; **152**: 2076–2083.
48. Caughey GH, Raymond WW, Bacci E, Lombardy RJ, Tidwell RR. Bis(5-amidino-2-benzimidazolyl)methane and related amidines are potent, reversible inhibitors of mast cell tryptases. *J Pharmacol Exp Ther* 1993; **264**: 676–682.
49. Ghosh SK, De Vos C, McIlroy I, Patel KR. Effect of cetirizine on exercise-induced asthma. *Thorax* 1991; **46**: 242–244.
